# Clinical Features, Short-Term Mortality, and Prognostic Risk Factors of Septic Patients Admitted to Internal Medicine Units: Results of an Italian Multicenter Prospective Study

**DOI:** 10.1097/MD.0000000000002124

**Published:** 2016-01-29

**Authors:** Antonino Mazzone, Francesco Dentali, Micaela La Regina, Emanuela Foglia, Maurizia Gambacorta, Elisabetta Garagiola, Giorgio Bonardi, Pierangelo Clerici, Ercole Concia, Fabrizio Colombo, Mauro Campanini

**Affiliations:** From the Internal Medicine Ward, Ospedale Civile, Legnano (AM, GB); Department of Clinical Medicine, Insubria University Varese (FD); Internal Medicine Hospital of La Spezia, La Spezia (MLR); Centre for Research on Health Economics, Social and Health Care Management—CREMS, University Carlo Cattaneo—LIUC, Castellanza (EF, EG); Internal Medicine Ward, Hospital Media Valle del Tevere, Todi (MG, PC); Microbiology Unit, Legnano Hospital, Legnano, Italy (PC), Italy; Infectious Diseases, Policlinico G.B. Rossi, University of Verona (EC); Internal Medicine Ward, Ospedale Niguarda Ca’ Granda, Milan (FC); and Internal Medicine Ward, Ospedale Maggiore della Carità, Novara, Italy (MC).

## Abstract

Only a few studies provided data on the clinical history of sepsis within internal Medicine units.

The aim of the study was to assess the short-term mortality and to evaluate the prognostic risk factors in a large cohort of septic patients treated in internal medicine units.

Thirty-one internal medicine units participated to the study. Within each participating unit, all admitted patients were screened for the presence of sepsis.

A total of 533 patients were included; 78 patients (14.6%, 95%CI 11.9, 18.0%) died during hospitalization; mortality rate was 5.5% (95% CI 3.1, 9.6%) in patients with nonsevere sepsis and 20.1% (95%CI 16.2, 28.8%) in patients with severe sepsis or septic shock. Severe sepsis or septic shock (OR 4.41, 95%CI 1.93, 10.05), immune system weakening (OR 2.10, 95%CI 1.12, 3.94), active solid cancer (OR 2.14, 95% CI 1.16, 3.94), and age (OR 1.03 per year, 95% CI 1.01, 1.06) were significantly associated with an increased mortality risk, whereas blood culture positive for *Escherichia coli* was significantly associated with a reduced mortality risk (OR 0.46, 95%CI 0.24, 0.88).

In-hospital mortality of septic patients treated in internal medicine units appeared similar to the mortality rate obtained in recent studies conducted in the ICU setting.

## INTRODUCTION

Sepsis is a frequent inflammatory disease with a high mortality and morbidity rate.^1^ Over the past few years, a number of different studies have reported an increasing incidence of this disease.^2–4^

Information on the epidemiology, causes, and management and prognosis of patients with sepsis came mainly from studies conducted within intensive care units (ICUs).^2,5,6^ However, a consistent number of studies suggest that a significant proportion of patients with sepsis (including those with severe sepsis) are admitted to internal medicine units, and not transferred to an ICU.^7,8^ Unfortunately only a few provided data on the epidemiology and management of sepsis in the internal medicine setting.^8^ Some studies have reported a higher mortality rate in patients with sepsis not admitted or with a delayed admission to an ICU.^9,10^ However, these studies were published before the implementation of recent international guidelines on the management of severe sepsis and septic shock.^6^ Thus, information on the clinical history of patients with sepsis treated outside an ICU is extremely limited.

Therefore, to address this knowledge gap, we conducted a perspective multicenter study, evaluating consecutive patients, with an objective diagnosis of sepsis treated in internal medicine units.

## METHODS

A protocol detailing specific objectives of the study and patients’ inclusion and exclusion criteria was developed by the Federation of Associations of Executives of Hospital Internists (FADOI) in collaboration with the Institute of Health and the University of Florence and the Centre for Research on Health Economics, Social and Health Care Management (CREMS), of University Carlo Cattaneo—LIUC.

At each of the participating centers, the ethics committee approved the study protocol and waived the need for informed consent.

### Patients

Thirty-one internal medicine units from 14 different Italian regions participated in the study (the complete list of participating centers is shown in Appendix 1). Consecutive patients with an objective diagnosis of sepsis, admitted to these units from March 1, 2012, to December 31, 2012, were included. A minimum of 15 patients was requested to each participating center and only adult patients with positive blood cultures were included. Patients transferred to the ICU from the emergency room were not included.

In each participating unit, all patients were actively screened for the presence of sepsis defined as a systemic inflammatory response secondary to infection (see “Definitions” below for detailed information). All the admitted patients who developed sepsis were initially enrolled. Patients in which blood cultures resulted negative were subsequently excluded from the study. Patients were followed up to discharge from the hospital to detect the presence of sepsis. Investigators in charge for the enrolment were physicians trained for the identification of patients with sepsis criteria. Although the use of most recent (at the time of the enrolment) available guidelines on this topic^11^ has been recommended, due to the observational nature of our study, decisions on the management and treatment of septic patients were left to the local investigators.

At the time of enrolment, the following data for each patient were collected: demographic characteristics (age, gender), reason for admission to the hospital, clinical presentation—including hemodynamic data (heart rate, blood pressure, temperature), presence of comorbidities, origin of primary infection (respiratory, gastrointestinal, genitourinary, or skin-muscle and primary bacteriemia), date of diagnosis of infection, and cultures performed, along with their results. Furthermore, information on arterial blood gases was gathered when available. Subsequently, patients were daily monitored until the end of hospitalization. Data on patients’ management (mechanical ventilation, vasoactive drugs, administration of drotrecogin alpha, dialysis) were recorded. Rate of transfer to the ICU, length of hospital stay, and in-hospital mortality were registered for all the patients. For each death, causes of death were collected.

Case report forms were prepared by the coordinating center (CREMS) and were sent to all participating subinvestigators. Local investigators were asked to fill out the form and then return it to the coordinating center. Detailed instructions explaining the aim of the study, instructions for data collection, and definitions for various items were available for all investigators before starting data collection. All data were cross-checked and centrally validated during the study. Errors or blank fields generated queries that were returned to each participating center for correction.

### Definitions

Infection was defined as the presence of a pathogenic micro-organism in a sterile milieu and/or clinically suspected infection, plus the administration of antibiotics.

As *Staphylococcus epidermidis* and *Staphylococcus hominis* may be skin contaminants, patients were considered as having this infection in the blood only when these 2 bacteria were isolated from 2 or more blood cultures drawn on separate occasions as commonly accepted.

According to the American College of Chest Physicians/Society of Critical Care Medicine Consensus Conference definitions, sepsis was defined as infection, plus 2 systemic inflammatory response syndrome criteria.^12^

Severe sepsis was defined as sepsis plus at least 1 organ dysfunction, except when that organ dysfunction already was present 48 h before the onset of sepsis.^13^ Septic shock was defined as sepsis plus either hypotension refractory to intravenous fluids (defined as persistent hypotension or a requirement for vasopressors after the administration of an intravenous fluid bolus) or hyperlactatemia.^13^ Organ dysfunction was defined in accordance with commonly established criteria.^1^

### Statistical Analysis

Continuous variables were expressed as mean, plus or minus the standard deviation (SD), or as median with minimum and maximum values when data did not have a normal distribution; categorical data are given as counts and percentages.

Percentage of admission for sepsis on the total number of admissions during the study period was calculated.

The mortality rate of the whole population during hospitalization and of patients with severe sepsis and with septic shock was expressed as percentage (with the corresponding 95% confidence intervals [CI] with continuity correction).

Characteristics of patients who died during hospitalization and who were discharged alive were compared. We used Student's *t* test or the Mann–Whitney test to compare continuous variables and the chi-square test or Fisher's exact test to compare proportions, as appropriate.

Subsequently, all the variables, statistically significant or marginally significant associated with an increased mortality risk during hospitalization at the univariate analysis, were introduced as covariates in a multivariate model (backward binary logistic regression model) with mortality as the dependent variable. To avoid false negative results, variables were introduced to the model when *P* < 0.20 at univariate analysis and removed from the model in the case of lack of significance. Results of multivariate analysis were presented as odds ratios (ORs) and corresponding 95% confidence intervals (CI). Two-tailed *P* values < 0.05 were used to indicate statistical significance for all the analyses. All the analyses were conducted using SPSS version 19.0 (SPSS Inc, Chicago, IL).

## RESULTS

**FIGURE 1:**
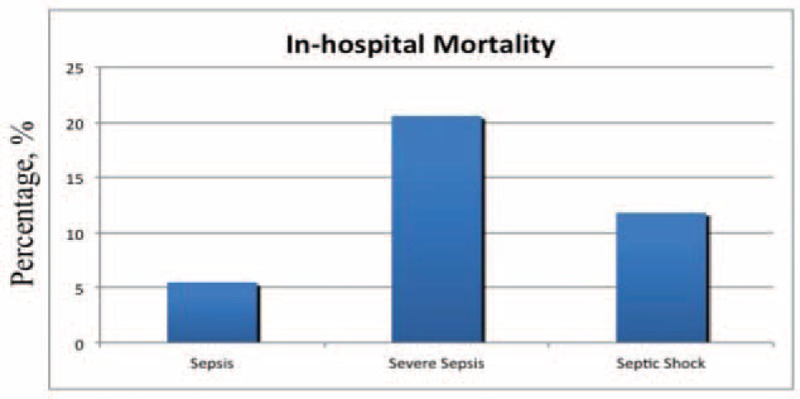
In-hospital mortality rate according to presentation of sepsis.

Baseline characteristics, clinical presentation, and principal comorbidities were summarized in Table 1. Main isolates at blood cultures were summarized in Table 2. Five hundred and thirty-three septic patients (mean age 73.3 years, 50.8% men) were included representing the 1.78% (95% CI 1.63, 1.95) of the admissions to the internal medicine units in the same period; 316 patients (59.3%) had a severe sepsis, and 17 (3.2.%) septic shock at the presentation. Sepsis most frequently came from genitourinary (30.8%), respiratory tract (26.5%), and gastrointestinal tract (18.9%), and 62 patients (11.6%) had a primary bacteriemia. Almost all the patients (94.2%) had at least 1 comorbidity: cardio or cerebrovascular disease (63.4%) and diabetes (30.8%) were the most frequent comorbidities. Antibiotic therapy was started within 1 h after clinical suspicion of sepsis in 337 patients (75.9%). There were 626 blood cultures positive: 313 (50.0%) for Gram-positive bacteria, 293 (46.8%) for Gram-negative bacteria, and only 20 (3.2%) for fungi. *Escherichia coli* (29.4%) was the most frequent isolated followed by *Staphylococcus aureus* (12.0%), *S. epidermidis* (12.0%), *Enterococcus faecalis* (7.4%), and *Klebsiella pneumoniae* (5.4%). In 497 cultures (79.4%) there was at least 1 antibiotic resistance and antibiotic resistance affected 44.7% of the empiric treatments. In particular, there were 88 ESBL producers and 22 MRSA. Seventy-eight patients (14.6%, 95% CI 11.9, 18.0%) died during hospitalization and 7 patients (1.3%) were transferred to the ICU; according to the clinical presentation mortality was 5.5% (95% CI 3.1, 9.6%) in patients with nonsevere sepsis, and 20.1% (95% CI 16.2, 28.8%) in patients with severe sepsis or septic shock (20.6%, 95% CI 16.5, 25.4% in patients with severe sepsis and 11.8%, 95% CI 3.3, 34.3% in patients with septic shock) (Figure 1).

**TABLE 1:**
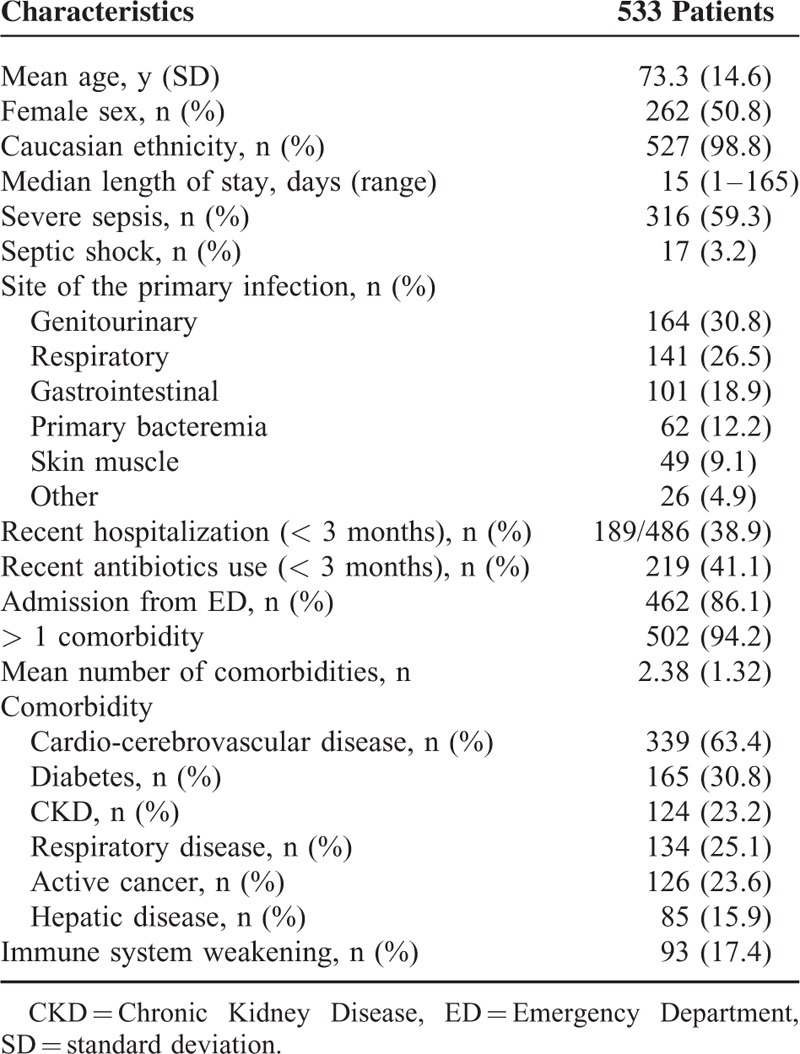
Baseline Characteristics

**TABLE 2:**
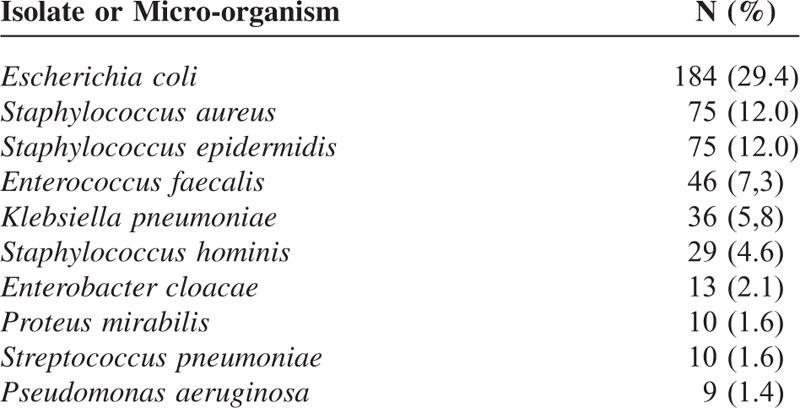
Main Isolates at Blood Cultures

As reported in Table 3, at the univariate analysis, female gender, older age, clinical presentation as severe sepsis or septic shock, presence of > 1 comorbidity, including active cancer, personal history of cardio or cerebrovascular disease, personal history of respiratory disease, and a state of immune system weakening, were significantly or marginally significantly associated with an increased mortality risk during hospitalization (*P* < 0.20 for all these variables). All the other variables evaluated were not significantly associated with an increased mortality risk (*P* > 0.20). Furthermore, when we consider the source of the primary infection, only origin from respiratory tract was marginally associated with an increased mortality risk (26/141, 18.4% vs 52/392 for other origins, 13.2%; *P = * 0.16) whereas all the other origins were not (data available upon request). Among the different bacteria isolated, blood culture positive for *E. faecalis* was significantly associated with an increased risk of death, whereas *E coli* infection was associated with a lower risk of death. Recent hospitalization was also associated with increased mortality risk at the univariate analysis, but information on this variable was available only from 486 patients (91.2% of the whole population).

**TABLE 3:**
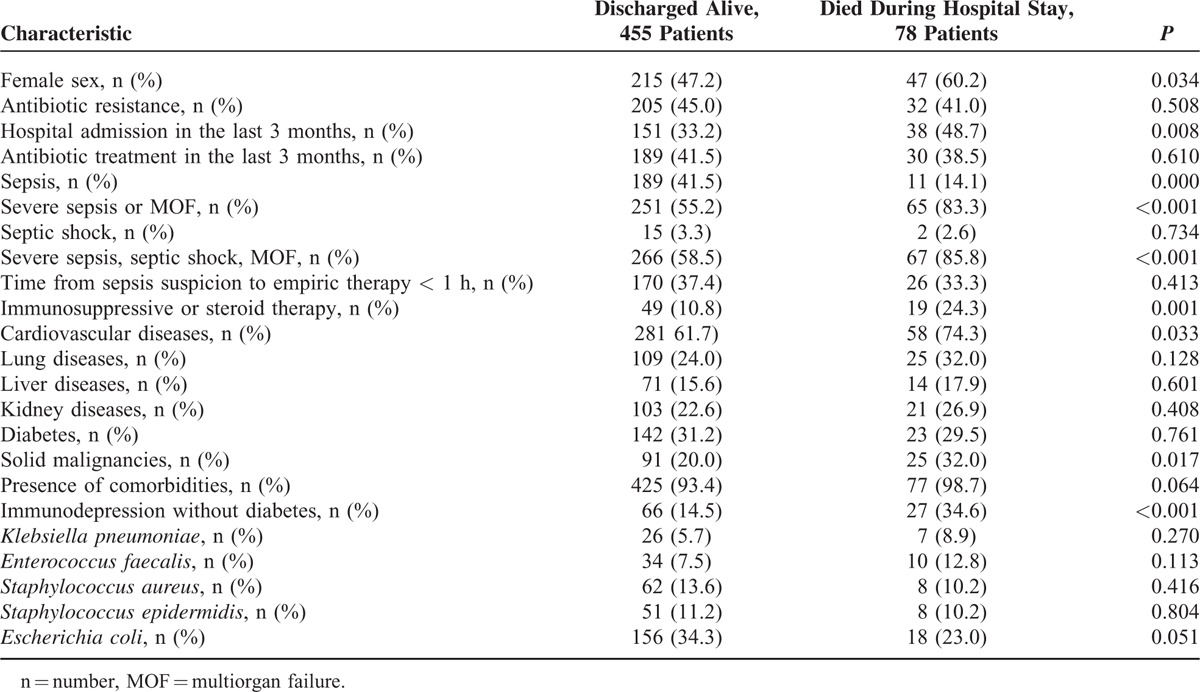
Results Univariate Analysis Comparing Characteristics of Patients Discharged Alive and Died During Hospitalization

**FIGURE 2:**
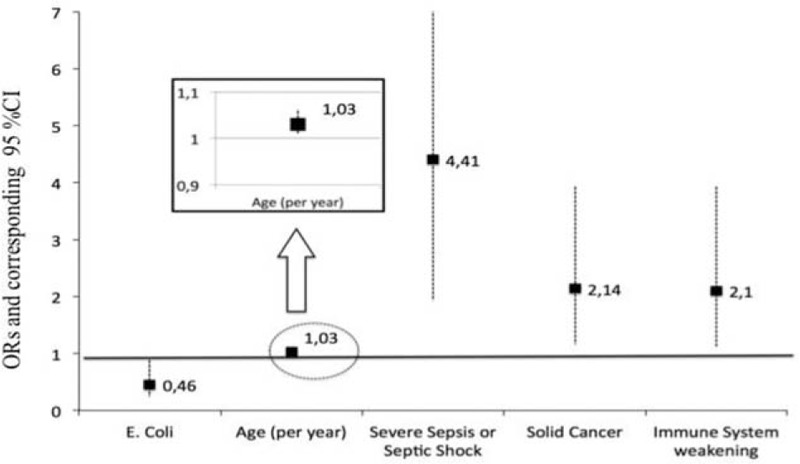
Independent predictors of in-hospital mortality at multivariate analysis. CI = confidence interval, ORs = odds ratios.

Using the backward binary logistic regression model, clinical presentation as severe sepsis or septic shock (OR 4.41 95% CI 1.93, 10.05), immune system weakening (OR 2.10, 95% CI 1.12, 3.94), presence of active solid cancer (OR 2.14, 95% CI 1.16, 3.94), and age (OR 1.03 per year, 95% CI 1.01, 1.06) were significantly associated with an increased mortality risk during hospitalization, whereas blood culture positive for *E. coli* was significantly associated with a reduced mortality risk (OR 0.46, 95% CI 0.24, 0.88) (Figure 2).

When the multivariate model was repeated, excluding recent hospitalization from the analysis, the results did not change (data not shown).

## DISCUSSION

Sepsis is an extremely frequent disease and its incidence continues to increase. The number of cases of severe sepsis in the United States only exceeds 750,000 per year,^1,14^ and it ranks tenth among the most frequent causes of death.^4^ In our study, we collected information on a quite large number of patients with sepsis and with positive blood culture who were consecutively admitted to an internal medicine unit. The in-hospital mortality of this population appeared to be in line with the results of recent studies that have evaluated the prognosis of patients with sepsis^14,15^ and that show a declining trend in the hospital mortality rate of these patients over the past few years.^16^ Our results may be of clinical relevance for clinicians as, to date, only a few studies have provided information on the epidemiology and clinical history of patients with sepsis diagnosed and treated outside an ICU.^8^ In fact, a significant proportion of patients with sepsis and even with severe sepsis are admitted to an internal medicine unit and not transferred to an ICU.^7^ Results of recent studies clearly show that the use of central venous catheterization to monitor central venous pressure and central venous oxygen saturation to guide the administration of intravenous fluids, vasopressors, packed red-cell transfusions, and dobutamine, did not modify the mortality and morbidity rate of patients with severe sepsis or septic shock.^17,18^ Thus, these results seem to suggest that patients may be treated safely, without the need of a continuous invasive monitoring, in a less intensive unit provided that an adequate therapy was rapidly administered.^19^ Interestingly, other recent studies conducted on patients with sepsis admitted to ICU showed similar results, confirming the validity of our findings.^20^ However, information on clinical and easily assessable factors, potentially associated with clinical deterioration and short-term mortality, remains critical as a delayed transfer to an ICU of these patients seems to be associated with a worse short-term prognosis.^20^

Besides the clinical presentation as severe sepsis or septic shock other factors such as the presence of active solid cancer or of immune system weakening and age appeared associated with an increased mortality risk during hospitalization. As no previous study has specifically assessed the role of potential risk factors for short-term mortality in patients with sepsis admitted to an internal medicine unit, it was not possible to compare our results with preceding literature in this field.

Information on the most frequent infection in different settings is crucial in order to establish the most appropriate treatment of patients with sepsis. Gram-positive bacteria were slightly more prevalent than Gram-negative bacteria (50.0% vs 46.8%) and only 3.2% of blood cultures were positive for fungi. *Escherichia coli* was the most frequent bacterium isolated followed by *S. aureus*, *S. epidermidis* and *E. faecalis* (7.4%). In previous studies, Gram-positive infections appeared more frequent than Gram-negative.^4^ However, more recently, in a study involving 14,000 ICU patients in 75 countries, Gram-negative bacteria were isolated in 62% of patients with severe sepsis with positive cultures, Gram-positive bacteria in 47%, and fungi in 19%.^5^

At least 1 antibiotic resistance was present in almost the 80% of the isolated at the blood culture and antibiotic resistance affected almost half of the empiric treatments. Interesting, *E. coli* infection was significantly associated with a reduced mortality risk in comparison to other bacteria at univariate analysis. *Escherichia coli* is very common in urinary tract infection sepsis that has a better prognosis in comparison to pneumonia-mediated sepsis.^5^ However, this association remained significant also at multivariate analysis that took in consideration among the other variables also the origin of infection. Finally, different from the results of a recent study on 327 adult septic patients admitted to ICU, primary bacteremia was not associated with an increased mortality risk in our population.^21^ Differences in the selection of the population may explain these different results. Alternatively, as only a small number of patients had a primary bacteremia in the 2 populations, these different results may be due to chance.

Our study has strengths and limitations. First, only patients with positive blood cultures were included in our study. Although this may be considered to be a selection and thus the patients may be not representative of general patients with sepsis admitted to an internal medicine unit, with this approach we were able to exclude all the patients without a clear diagnosis of sepsis, increasing the validity of our results. Second, information on recent hospitalization was not available in almost 9% of included patients. Although the lack of this information in a not negligible proportion of patients may affect the validity of our results, however the repetition of the multivariate analysis after the exclusion of this parameter gave similar results minimizing this possibility. In addition, <1% of data of all the other were missing. Third, results on the mortality rate of the subgroup of patients with septic shock should be interpreted with extreme caution as an extremely low number of patients with this condition (n = 17) have been included in our study. Finally, there may be important differences in the epidemiology, management and clinical history of sepsis among the involved centers. However, due to the relative low number of patients included in each center, this could not be accurately explored.

## CONCLUSIONS

In conclusion, the in-hospital mortality rate of septic patients admitted to internal medicine units appeared in line with recent reports of the literature in ICU setting. Besides the clinical presentation as severe sepsis or septic shock, other factors appeared to be helpful in defining the short-term prognosis of septic patients and may be used to define the adequate management of these patients. However, other larger prospective studies are warranted to confirm our preliminary findings.
